# Comparative proteome analysis reveals conserved and specific adaptation patterns of *Staphylococcus aureus* after internalization by different types of human non-professional phagocytic host cells

**DOI:** 10.3389/fmicb.2014.00392

**Published:** 2014-08-01

**Authors:** Kristin Surmann, Stephan Michalik, Petra Hildebrandt, Philipp Gierok, Maren Depke, Lars Brinkmann, Jörg Bernhardt, Manuela G. Salazar, Zhi Sun, David Shteynberg, Ulrike Kusebauch, Robert L. Moritz, Bernd Wollscheid, Michael Lalk, Uwe Völker, Frank Schmidt

**Affiliations:** ^1^Interfaculty Institute for Genetics and Functional Genomics, University Medicine GreifswaldGreifswald, Germany; ^2^ZIK-FunGene Junior Research Group Applied Proteomics, Interfaculty Institute for Genetics and Functional Genomics, University Medicine GreifswaldGreifswald, Germany; ^3^Institute of Biochemistry, Ernst-Moritz-Arndt-University GreifswaldGreifswald, Germany; ^4^Institute for Microbiology, Ernst-Moritz-Arndt-University GreifswaldGreifswald, Germany; ^5^Institute for Systems BiologySeattle, WA USA; ^6^Institute of Molecular Systems Biology, ETH ZurichZurich, Switzerland

**Keywords:** *Staphylococcus aureus*, human cell lines, host-pathogen interaction, proteomics, label-free quantitation

## Abstract

*Staphylococcus aureus* is a human pathogen that can cause a wide range of diseases. Although formerly regarded as extracellular pathogen, it has been shown that *S. aureus* can also be internalized by host cells and persist within these cells. In the present study, we comparatively analyzed survival and physiological adaptation of *S. aureus* HG001 after internalization by two human lung epithelial cell lines (S9 and A549), and human embryonic kidney cells (HEK 293). Combining enrichment of bacteria from host-pathogen assays by cell sorting and quantitation of the pathogen's proteome by mass spectrometry we characterized *S. aureus* adaptation during the initial phase between 2.5 h and 6.5 h post-infection. Starting with about 2 × 10^6^ bacteria, roughly 1450 *S. aureus* proteins, including virulence factors and metabolic enzymes were identified by spectral comparison and classical database searches. Most of the bacterial adaptation reactions, such as decreased levels of ribosomal proteins and metabolic enzymes or increased amounts of proteins involved in arginine and lysine biosynthesis, enzymes coding for terminal oxidases and stress responsive proteins or activation of the sigma factor SigB were observed after internalization into any of the three cell lines studied. However, differences were noted in central carbon metabolism including regulation of fermentation and threonine degradation. Since these differences coincided with different intracellular growth behavior, complementary profiling of the metabolome of the different non-infected host cell types was performed. This revealed similar levels of intracellular glucose but host cell specific differences in the amounts of amino acids such as glycine, threonine or glutamate. With this comparative study we provide an impression of the common and specific features of the adaptation of *S. aureus* HG001 to specific host cell environments as a starting point for follow-up studies with different strain isolates and regulatory mutants.

## Introduction

The Gram-positive bacterium *Staphylococcus aureus* is known to be a commensal and colonizes 20–30% of the human population without causing any symptoms. However, it is also the cause of many infectious diseases in humans ranging from mild skin infections to toxin-mediated diseases like the toxic shock syndrome as well as endocarditis, pneumonia, and septicaemia (Lowy, 1998; Wertheim et al., 2005). Though formerly regarded to be an extracellular pathogen it has also been shown that *S. aureus* is able to invade, persist and replicate inside non-professional phagocytotic cells (Hudson et al., 1995; Almeida et al., 1996; Garzoni and Kelley, 2009; Tuchscherr et al., 2011). Furthermore, *S. aureus* infects different types of organs and cells (Wertheim et al., 2005; Haslinger-Löffler et al., 2006).

Adaptation of *S. aureus* to the intracellular environment of host cells likely requires complex changes in transcription, protein synthesis and metabolism which in principle can all be captured comprehensively by integrated OMICs approaches. However, such *in vivo* OMICs are hampered by the limited availability of either bacterial mRNA or proteins from such settings. Thus, despite their great potential, only a few transcriptome or proteome studies have so far been performed to investigate the adaptation of *S. aureus* to the host environment following internalization.

Analyzing the transcriptome of *S. aureus* 6850 in the initial hours after internalization by A549 cells Garzoni et al. observed profound transcriptional adaptation patterns that involved approximately 40% of all putative ORFs of *S. aureus* 6850 (Garzoni et al., 2007). In another approach the transcriptome profiles of methicillin-resistant *S. aureus* USA300 were investigated in human abscesses and infected mouse kidneys (Date et al., 2014). That study established that transcriptional adaptations in both models were remarkably similar and included up-regulation of genes coding for multiple proteases and toxins as well as iron and peptide transporters. Furthermore, the specific influence of the global regulators *agrB* and *saeRS* on regulation of virulence associated genes *in vivo* was examined.

Proteome studies are even more challenging because the large excess of interfering human host proteins complicates identification especially of low abundant bacterial proteins. In a first attempt to investigate the adaptation of *S. aureus* to host cells at the proteome level Miller et al. analyzed the impact of co-cultivation with THP-1 macrophages but confined the study to non-adherent, non-internalized *S. aureus* cells (Miller et al., 2011). The comparative study revealed that *S. aureus* NCTC8325 induced the stringent response, activated the alternative sigma factor SigB, enhanced its capability to respond to oxidative stress and produced increased levels of virulence factors including phenol-soluble modulins (PSMs) in response to the presence of macrophages (Miller et al., 2011). Proteome analysis of internalized bacteria requires enrichment by differential centrifugation, immunomagnetic separation, or fluorescent assisted cell sorting. A first analysis of the complex proteome adaptation of *S. aureus* to the intracellular milieu of human epithelial cells was performed in 2010 (Schmidt et al., 2010). Human bronchial epithelial S9 cells were infected with *S. aureus* HG001 pMV158GFP, which expresses green fluorescent protein constitutively, allowing separation of internalized *S. aureus* from the whole cell lysate by cell sorting prior to LC-MS/MS analysis of the bacterial proteins (Schmidt et al., 2010). In that study, 591 bacterial proteins were identified and 367 could be quantified using stable isotope labeling of amino acids in cell culture (Ong et al., 2002). Using an optimized variant of the original protocol we were now able to identify 1302 bacterial proteins and monitor more than 980 *S. aureus* proteins quantitatively from as little as 1–2 million internalized bacteria (Pförtner et al., 2014). Such a comparative profiling of the *S. aureus* wild type HG001 and its isogenic Δ*sigB* mutant revealed that the alternative sigma factor SigB of *S. aureus* is activated immediately following internalization into S9 cells and that loss of SigB triggered proteome changes reflecting the different residual growth rates of wild type and *SigB* mutant, respectively, the resistance to methicillin, adaptation to oxidative stress and protein quality control mechanisms (Pförtner et al., 2014).

However, the particular response of bacterial pathogens will also depend on the host responses mounted and thus on the host cell line used. Recently, Eisenreich et al. reviewed the variability of host responses either of different cell lines infected with the same pathogen or the same cell line infected with different pathogens (Eisenreich et al., 2013). The data strongly support the notion that the choice of the host cell line or organism crucially influences the impact of bacterial infections. To our knowledge, the variation of adaptive responses of *S. aureus* upon internalization by different host cells has not been addressed yet.

Here, we comparatively analyzed intracellular survival and physiological adaptation of *S. aureus* HG001 to two commonly used human lung epithelial cell lines, A549 (Liang et al., 2009; Wang et al., 2013), and S9 cells (Below et al., 2009; Hermann et al., 2014). Additionally, we used human embryonic kidney HEK 293 cells. This cell line was chosen since it is also an established cell culture model for infections with *S. aureus* (Sinha et al., 1999; Cucarella et al., 2002; Maya et al., 2012). We characterized the proteome adaptation of *S. aureus* HG001 to the three different cell lines during the initial phase between 2.5 h and 6.5 h post-infection (p.i.). Analyzing each 2 × 10^6^ bacteria, 1443 *S. aureus* proteins, including metabolic enzymes and virulence factors, were identified and quantified by a combination of highly sensitive LC-MS/MS approaches, spectral library and classical FASTA database searches in all three cell lines. Thus, analyzing three biologically independent sample series we were able to cover almost 50% of the total proteome of *S. aureus* HG001 and describe common and specific adaptation processes of *S. aureus* upon internalization by different cell lines. Furthermore, we generated complementing metabolome data for each host cell line representing the nutrient supply available for the bacteria in each cell line.

## Materials and methods

### Bacterial strain and cultivation conditions

*S. aureus* strain HG001 (Herbert et al., 2010) carrying plasmid pMV158GFP (Nieto and Espinosa, 2003) was used throughout the study. Bacteria were cultivated in prokaryotic minimal essential medium (pMEM) until the exponential growth phase (OD_600_ 0.4) as described previously (Depke et al., 2014). The number of bacterial cells present in the culture at OD_600_ was determined by flow cytometry with a Guava easyCyte™ flow cytometer (Millipore, Billerica, MA, USA) using GFP constitutively expressed from pMV158GFP.

### Cell lines and cultivation conditions

S9 cells [adeno 12 SV40 hybrid virus-transformed human bronchial epithelial cells, ATCC® number CRL-2778 (Zeitlin et al., 1991; Flotte et al., 1993)] and A549 cells [human lung cancer-derived alveolar epithelial cells, ATCC® number CCL-185, (Lieber et al., 1976)] were cultivated in eukaryotic minimal essential medium (eMEM) as described previously (Pförtner et al., 2013). HEK 293 cells [adenovirus type 5-transformed human embryonic kidney cells (Graham et al., 1977)] were cultivated in modified eMEM for HEK 293 cells (HEK-eMEM; 1×MEM [Biochrom AG), supplemented with additional 10% (v/v) dialyzed fetal bovine serum (FBS, Invitrogen), 2% (v/v) L-glutamine (PAA), 1% (v/v) sodium pyruvate (PAA), and 1% (v/v) non-essential amino acids (PAA)]. Cells were cultured in 10-cm-diameter tissue culture plates at 37°C, 5% CO_2_ in a humid atmosphere. Prior to infection experiments, host cells were seeded in 24-well tissue culture plates 3 days in advance and cultivated until confluence.

### Internalization settings

*S. aureus* HG001 pMV158GFP was cultivated in pMEM until OD_600_ 0.4. Based on a calibration curve, an infection mix of bacterial culture was prepared in host cell medium buffered with 2.2 g/L sodium hydrogen carbonate. Bacterial cultures were diluted in this mix to infect host cells with a multiplicity of infection (MOI) of 25. Afterwards, the cell culture medium of confluent host cells was replaced by this infection mix, and bacteria could sediment, attach to and be internalized by host cells in an incubator at 37°C and 5% CO_2_ for 1 h. Subsequently, one mL of host cell supernatant containing non-internalized bacteria was collected to represent the non-adherent control. The remaining infection mix was replaced by host cell medium containing 10 μg/mL lysostaphin (AMBI PRODUCTS LLC, Lawrence, NY, USA). Non-internalized *S. aureus* cells were killed efficiently in this step within 30 min (Pförtner et al., 2013). After 2.5 h and 6.5 h supernatant was removed and cell layers were washed twice with PBS. Host cells were lysed with 0.1% Triton X-100 in water for 5 min at 37°C and rinsed afterwards with PBS. Bacterial cell counts were again determined with a Guava easyCyte™ flow cytometer (Millipore).

### Cell sorting and on-membrane digestion

GFP expressing bacteria from exponentially growing cultures (OD_600_ 0.4), non-adherent controls, and internalized bacteria 2.5 h and 6.5 h p.i. were sorted from culture or host cell debris using a FACSAria II onto a low protein binding filter membrane (0.22 μm pore size) of a 96-well microtiter plate (Millipore, Schwalbach, Germany) by applying vacuum (450–550 mbar) to the filter plate to allow constant removal of the fluid (Pförtner et al., 2013). The 488 nm laser was applied to excite GFP and emitted fluorescence was detected at 515–545 nm (FITC-channel). Two million bacteria were collected per sample. Filters were rinsed with 200 μL PBS, and membranes were cut in four pieces and stored at −20°C. On-membrane digestion was performed as described earlier using lysostaphin and the protease trypsin and subsequent purification of peptides using C_18_ ZipTip columns (Merck Millipore, Billerica, MA, USA) (Pförtner et al., 2014).

### Analysis of bacterial proteins by mass spectrometry and subsequent data analysis

MS analysis was performed on a Q Exactive mass spectrometer (Thermo Fisher Scientific, Waltham, MA, USA) coupled to a TriVersa NanoMate (Advion, Ltd., Harlow, UK) after separation of peptides with a Dionex UltiMate 3000 nano-LC system (Dionex/Thermo Fisher Scientific, Idstein, Germany). Peptides were separated on a 25 cm analytical column packed with 2 μm C18 particles (Acclaim PepMap RSLC, Thermo Scientific) with the help of a linear gradient ranging from 2 to 25% buffer (0.1% (v/v) acetic acid in acetonitrile). MS data were acquired with a MS scan resolution of 70,000, and the 10 most abundant isotope patterns with charge state ≥2 from the survey scan were subjected to MS/MS analysis with a resolution of 17,5000. Fragmentation was achieved using higher energy collisional dissociation (HCD). Further details are available as Supplementary Material.

Resulting raw data files were converted to mzML format using msconvert. Then, the mzML files were searched using COMET (Eng et al., 2013) and SpectraST (Lam et al., 2007) and processed using Trans-Proteomic Pipeline (Keller and Shteynberg, 2011). The database contained 84,911 human protein entries [complete proteome and VARSPLIC (Kersey et al., 2000)] and 2891 sequences for *S. aureus*. Common contaminants (115 cRAP) and a sequence-shuffled decoy counterpart were added to the database. For COMET, the parent mass error was set to ± 50 ppm. N-terminal protein acetylation and methionine oxidation were set as variable modifications. The maximum number of missed cleavage sites was set to 2, and number of enzyme termini was set to 1. For SpectraST, the parent mass error was set to ± 1.0 Daltons. The spectral library was constructed from previous Q Exactive runs which were searched using COMET with the same parameters using a *S. aureus* protein database only [Michalik et al., unpublished data]. Peptides identified with iProphet probability ≥0.9 were used to construct the spectral library (Shteynberg et al., 2011). The PeptideProphet outputs from both search engines were combined using iProphet.

The reSpect algorithm was applied to identify and attenuate the peaks in the MS/MS spectra that were excluded by the first pass search. A second search round was performed on the reSpect processed spectra, with a mass tolerance matching the selection window of the mass spectrometer and using possible charge states of 1 through 5, which allowed the identification of novel distinct peptide sequences not seen in the single pass analysis. The reSpect searches were analyzed separately from the first pass searches, and also using PeptideProphet and iProphet to establish accurate error rates.

Only peptides with a probability greater than 0.8 (~ TPP error rate < 0.01) and without missed cleavages were considered for further relative quantitation on protein level. The quantitation was performed using the “MSstats R package for statistical relative quantitation of proteins and peptides” implemented in Skyline package v2.5 (Schilling et al., 2012; Choi et al., 2013). The areas under the curve (AUC) of peptides were summed to obtain single protein intensities. Mean values from three biological replicates were used which were median normalized to the values of non-adherent bacteria control.

The mass spectrometry proteomics data have been deposited to the ProteomeXchange Consortium (Vizcaino et al., 2014) via the PRIDE partner repository with the dataset identifier PXD001003.

Principal component analysis was performed using the Genedata Analyst v7.6 software (Genedata AG, Basel, Switzerland). Median normalized log_10_ intensity values for all proteins quantified in all cell lines were used for calculation of the variances of the cell lines and sampling points. For each time point data from three independent biological samples were used to calculate average values.

Box blots of functional groups were generated using the SEED database of *S. aureus* NCTC8325 [SEED DB version 2.0 (Overbeek et al., 2005)], and Voronoi treemaps were created using the Paver software (DECODON GmbH) (Bernhardt et al., 2013).

### Analysis of host cell metabolome

#### Sampling and extraction of host cell metabolites

Confluent cells in 15-cm-diameter tissue culture plates were treated for 1 h with a sterile “infection mix” as described above, imitating a MOI 25 but leaving out bacteria. For this control the pH was set to 6.9, corresponding to the pH of a bacterial culture at OD_600_ 0.4. Samples of cellular extracts were generated and extracted as described previously (Gierok et al., 2014). In brief, the supernatant was collected separately. After washing with physiological sodium chloride solution ice cold methanol was added to the cells which were subsequently scraped from the plate and transferred into a centrifugation tube. The plate was washed with double distilled water, which was combined with the methanol fraction and frozen in liquid nitrogen. After adding of the internal standards, metabolites were extracted with a methanol:water:chloroform ratio of 5:5:1 and samples were prepared for analytical measurements as described (Gierok et al., 2014).

#### Acquisition of metabolome data

***GC/MS setup and analysis***. Lyophilized samples were derivatized using a two-step derivatization method with MeOX (Sigma-Aldrich) and MSTFA (Chromatographie Service GmbH) (Strelkov et al., 2004). For identification and quantification of metabolites a GC/MS method described previously was applied (Gierok et al., 2014). Qualitative and quantitative analyses were performed using ChromaTOF software (LECO Corporation). The computed metabolite concentrations were further related to the respective cell number.

***LC/MS setup and analysis***. For identification and quantitation of metabolites a LC/MS method with an ion-pairing reagent and a SymmetryShield RP18 column (Waters) was used with a setup as described (Gierok et al., 2014). Metabolite quantification was performed by QuantAnalysis® (Bruker Daltonik) using previously acquired standard curves. The computed metabolite concentrations were further normalized and related to the respective cell number.

***^1^H-NMR analysis of the extracellular medium***. Two mL of the medium were filtered through a 0.22 μm syringe sterile filter (Sarstedt AG&Co) and directly frozen. Qualitative and quantitative data analysis was carried out in a Bruker AVANCE-II 600 NMR spectrometer operated by TOPSPIN 3.1 software and by using AMIX® (Bruker Biospin) as described (Dörries and Lalk, 2013). The NMR-data are available via the MetaboLights database (Haug et al., 2013) under the accession number MTBLS102 (www.ebi.ac.uk/metabolights/MTBLS102).

## Results and discussion

### Experimental setup and analysis workflow

It is well established that different cell lines exhibit different protein patterns and adaptation responses that are tailored to their specific physiological role within the human body (Schirle et al., 2003). Therefore, it is of interest to comparatively profile bacterial adaptation after internalization by different cell line types such as S9, A549, and HEK 293. *S. aureus* HG001 was grown until OD_600_ 0.4 and transferred to human cell lines grown to confluence. After 2.5 h and 6.5 h the internalized bacteria were isolated via cell sorting (Pförtner et al., 2014) and subjected to analysis by mass spectrometry. Time resolved proteome patterns of internalized *S. aureus* HG001 were compared with the protein profiles of the non-adherent control bacteria that were exposed to human host cells but were not internalized. This control was chosen because previous transcriptome studies have established that *S. aureus* only mounts adaptation reactions after internalization but not after mere contact with human non-professional phagocytic cells (Garzoni et al., 2007).

**Figure 1:**
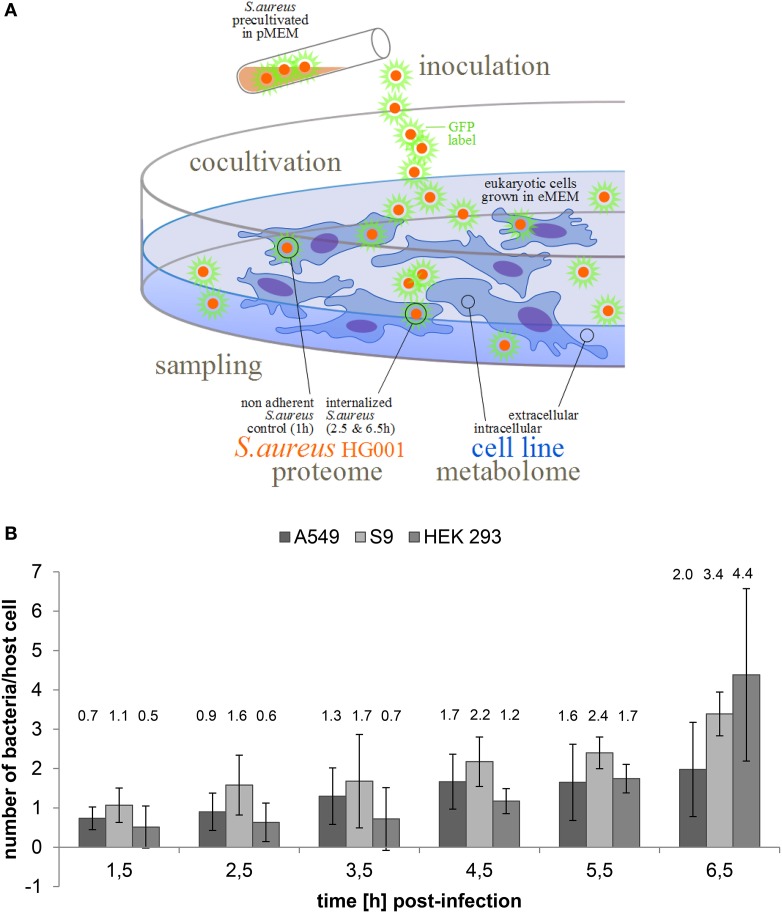
**Internalization of *S. aureus* HG001 by human cell lines. (A)** Experimental course of action. GFP expressing bacteria were cultivated in pMEM until OD_600_ 0.4 (exponential growth). Human cell lines (S9, A549, and HEK 293) were cultivated in eMEM and infected with *S. aureus* HG001 pMV158GFP cells. The proteome of the non-adherent and the internalized bacteria was investigated after cell sorting via flow cytometry. In parallel, the metabolome of non-infected human cells and the cell culture supernatant was recorded. **(B)** Number of internalized bacteria per host cell. GFP expressing bacteria were counted by flow cytometry from lysed host cells. For all three cell lines time-resolved bacterial counts are provided. Results represent the mean and standard deviation of three biological replicates.

In order to support the proteome data we further investigated the metabolome profile of the human host cells and the corresponding culture supernatant to elucidate metabolic shifts and available nutrients (Figure 1A). Whereas the supernatants of all three human cell lines displayed similar metabolite profiles, some metabolites were detected in different amounts in the intracellular metabolome of the three cell lines (Supplementary Material Figure 1).

### Intracellular survival and replication

Fluorescence microscopy and flow cytometry revealed an intracellular replication of *S. aureus* in all three cell lines as well as formation of clusters at 6.5 h p.i. (Supplementary Material Figure 2). In all three cell lines, only a rather small proportion of host cells ranging from 10–20% was indeed infected at an MOI of 25. Intracellular growth of *S. aureus* HG001 differed when internalized by different cell types (Figure 1B). The number of intracellular bacteria started to increase immediately after internalization by A549 and S9 cells and was finally approximately threefold higher 6.5 h p.i.. In contrast, numbers of internalized bacteria remained almost constant for up to 3.5 h p.i. in HEK 293 and then increased more rapidly in the subsequent 3 h to finally reach a level almost eightfold higher than 1.5 h p.i. (Figure 1B). Considering these values, we assumed that the different growth behavior might be reflected in the proteome adaptation of *S. aureus* HG001 upon internalization.

### Proteome analysis of internalized *S. aureus*

#### Staphylococcal protein identification and quantitation

**Figure 2:**
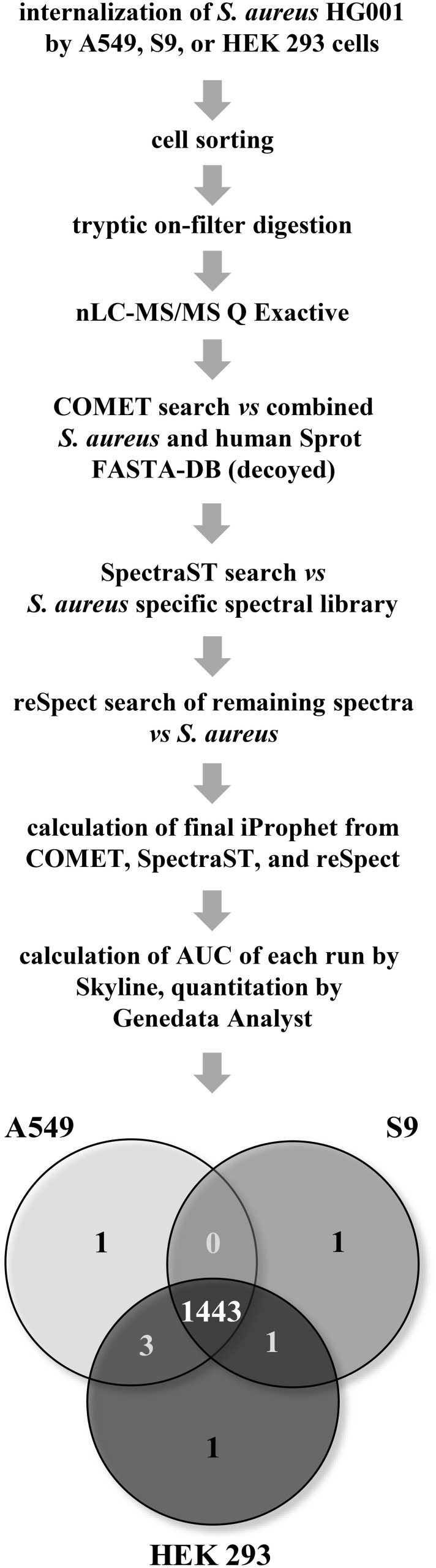
**Proteomics identification and quantitation workflow**. Bacteria were sorted from host cell debris onto membrane filters by flow cytometry. Subsequently, bacterial proteins were digested on-filter using trypsin prior to nLC-MS/MS acquisition with a Q Exactive. Database analysis was performed in three steps: (i) COMET search against human and *S. aureus* HG001 Sprot database, (ii) SpectraST against a specific *S. aureus* HG001 spectral library, and (iii) reSpect search of non-matching spectra. Calculation of AUC of peptides and proteins exceeding an iProphet score of 0.8 was performed with Skyline and the Genedata Analyst. The Venn diagram represents all proteins which were detected in any of the three biological replicates for each host cell line.

The main limitation of host-pathogen proteomics approaches is the low number of available bacteria and the large excess of human host proteins. Due to this fact, it is still a challenge to reliably identify *S. aureus* specific peptides in such a complex mixture. That is why we first applied a COMET search using a decoy database of *S. aureus* HG001 combined with human Uniprot/Swissprot DB and identified 1393 staphylococcal proteins in total with a peptide probability greater than 0.8 (~ FDR < 0.01). Finally, a spectral library comparison using a *S. aureus* HG001 specific database [Depke et al., unpublished data] complemented with a reSpect search was applied (Figure 2). Taken all identifications together, 1484 proteins were finally identified with an FDR less than 0.01.

With respect to quantitation, 1443 staphylococcal proteins could also be quantified over time for all three cell line models (Figure 2, Supplementary Material Table 1). With the improved settings in both mass spectrometry and data analysis we were now able to quantify about 50% of the whole proteome (2891 proteins in *S. aureus* NCTC8325 database) from 2 million *S. aureus* HG001 cells after internalization and therefore provide a comprehensive description of the behavior of this important pathogen during infection. This is roughly three times more compared to a first proteome study of internalized *S. aureus* HG001 (Schmidt et al., 2010).

**Figure 3:**
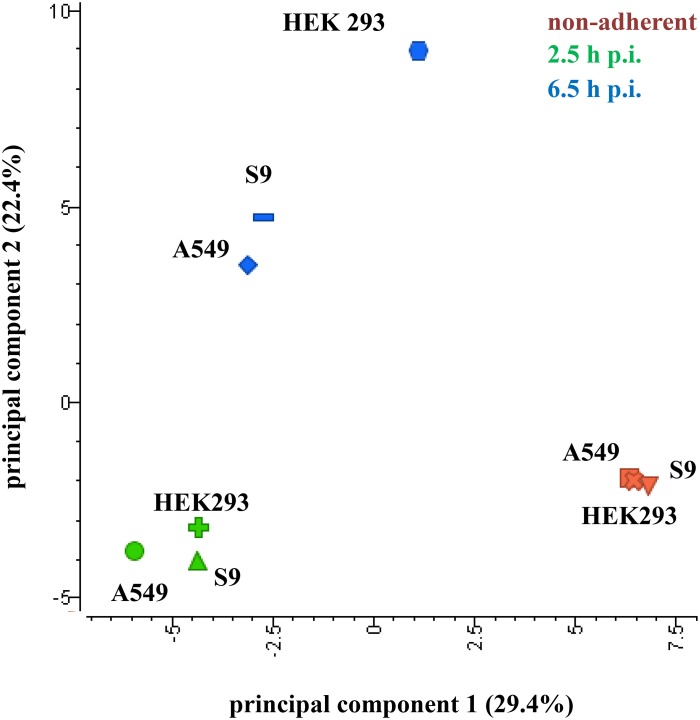
**Principal component analysis of *S. aureus* proteins**. Samples were separated by the cell lines (component 1, 29.4% variance) as well as the different treatment groups (component 2, 22.4% variance). Only proteins with quantitative data for all samples, (median normalized AUC data, probability >0.8) were considered for the analysis. Data from three biological replicates are represented.

**Figure 4:**
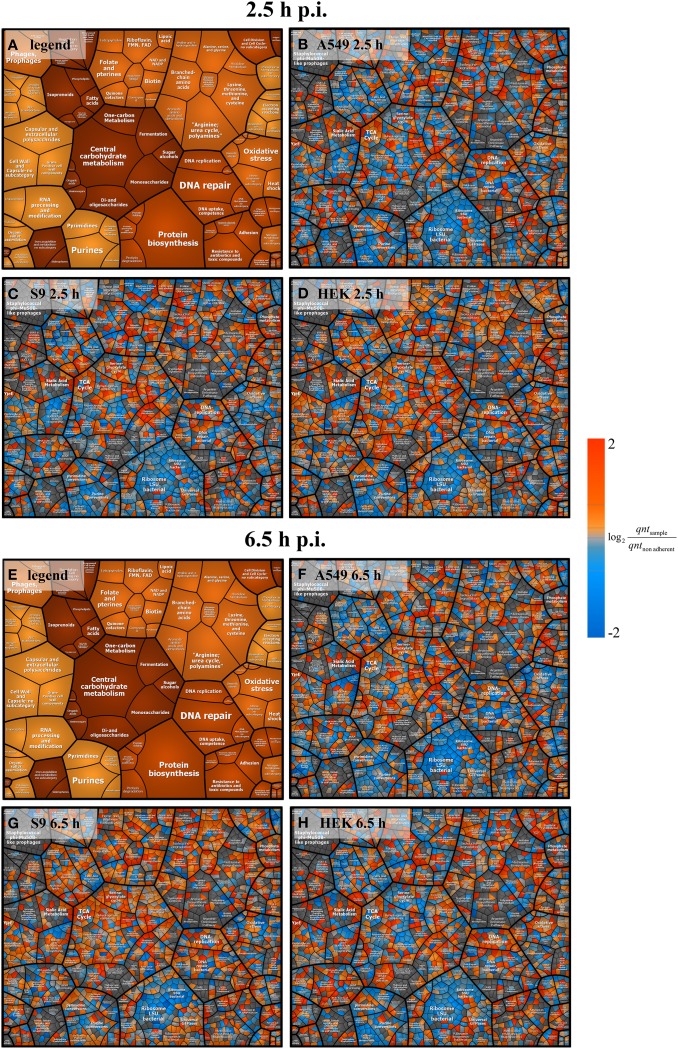
**Voronoi treemap analysis of *S. aureus* proteins**. Ratios from intensity values 2.5 h and 6.5 h p.i. compared to the non-adherent control are depicted. All pictures represent data on protein level clustered by pathways. Panels **(A,E)** serve as a legend showing only the pathways. Data at 2.5 h p.i. are represented in panel **(B)** (A549 cells), panel **(C)** (S9 cells), and panel **(D)** (HEK 293 cells). Data at 6.5 h p.i. are shown in panel **(F)** (A549 cells), panel **(G)** (S9 cells), and panel **(H)** (HEK 293 cells). Blue spots indicate lower levels in the internalized bacteria compared to the non-adherent control; red colors represent higher levels of proteins in response to internalization compared to the non-adherent control. Average values from three independent biological samples are displayed.

In order to gain a first inside into the global response of *S. aureus* to internalization by three different host cell lines a principal component analysis (PCA) plot of three sampling points (non-adherent control, 2.5 h and 6.5 h p.i.) was generated (Figure 3). For this analysis, the median-normalized data of all quantified proteins were taken into account. All samples from internalized bacteria, both at 2.5 h and 6.5 h p.i., were clearly separated from the non-adherent controls by the first component. Remarkably, the protein pattern of *S. aureus* internalized by HEK 293 kidney cells for 6.5 h was clearly separated in the second component from the other two *S. aureus* samples internalized by epithelial cell lines for the same time period, which indicates a different behavior of *S. aureus* inside the HEK 293 cells. The Voronoi treemaps displayed in Figure 4 provide a protein-resolved picture of the proteome adaptation where proteins which are assigned to the same biochemical pathway or adaptation reaction are grouped together (legends in Figures 4A,E). Again the patterns of *S. aureus* internalized by epithelial cells (S9 or A459) look more similar to each other than to HEK293-internalized *S. aureus*. Exemplarily one can focus on protein biosynthesis which appears more reduced in level in comparison to the control in *S. aureus* originating from A549 (Figure 4B) and S9 (Figure 4C) compared to those isolated 2.5 p.i. from HEK 293 cells (Figure 4D). On the contrary, proteins involved in central carbon metabolism tend to be in general more strongly induced in *S. aureus* from A549 (Figure 4F) and S9 (Figure 4G) cells than in *S. aureus* from HEK 293 cells 6.5 h p.i. (Figure 4H).

#### Common reaction of *S. aureus* proteome to internalization by three different host cells

In order to characterize *S. aureus* specific pathways or reactions which were similar after internalization by the three different host cell lines, we plotted the log_2_ intensities of the proteins belonging to these pathways in a box-plot. For this analysis, the third level of the functional category level from SEED was used (Supplementary Material Table 2) which combines several proteins which participate in the same functional pathways and is thus more robust than analysis at the level of individual proteins.

**Figure 5:**
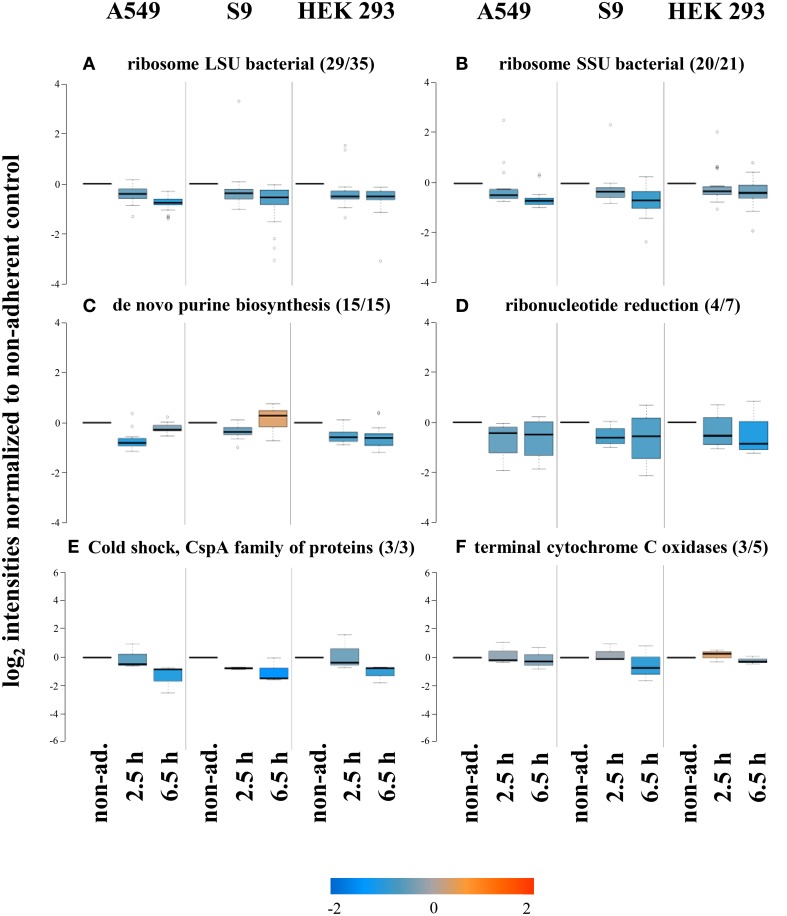
**Protein groups displaying reduced levels after internalization by all three cell lines**. After internalization some proteins were found in decreased levels, among them many groups required for growth such as ribosomal proteins **(A,B)**, and *de novo* purine biosynthesis **(C)**. Also proteins involved in ribonucleotide reduction **(D)**, cold shock adaptation **(E)**, and those representing terminal cytochrome C oxidases **(F)** were reduced upon internalization. Average values of log_2_ intensities from three biological replicates each for non-adherent bacteria as well as 2.5 h and 6.5 h p.i. are presented. Blue spots indicate lower levels in the internalized bacteria compared to the non-adherent control, red colors represent higher levels of proteins in response to internalization compared to the non-adherent control.

Presumably, *S. aureus* displays significantly reduced growth rates after internalization by host cells compared to those possible in the cell culture medium alone. In agreement with this assumption we observed decreased levels of proteins composing the large (ribosome LSU bacterial, 29 of 35, Figure 5A) and small (ribosome SSU bacterial, 20 of 21, Figure 5B) subunit of the ribosome during the first 6.5 h of internalization into all three cell lines. Furthermore, proteins involved in *de novo* purine biosynthesis (15 of 15, Figure 5C) and ribonucleotide reduction (4 of 7, Figure 5D) were also decreased in level probably reflecting the lower levels required to sustain lower growth rates. The level of cold shock proteins (Figure 5E) was also commonly reduced after internalization compared to that in non-adherent control cells, but the physiological rationale for this observation is currently not clear.

Interestingly, the level of components of the cytochrome C oxidase (3 of 5) was also reduced in internalized bacteria vs. the non-adherent control (Figure 5F). This regulation possibly indicates an adaptation to reduced oxygen levels inside host cells, because this main terminal oxidase is preferentially used under aerobic conditions (Götz and Mayer, 2013) and likely replaced by a specialized oxidase under the likely microaerobic conditions inside host cells.

**Figure 6:**
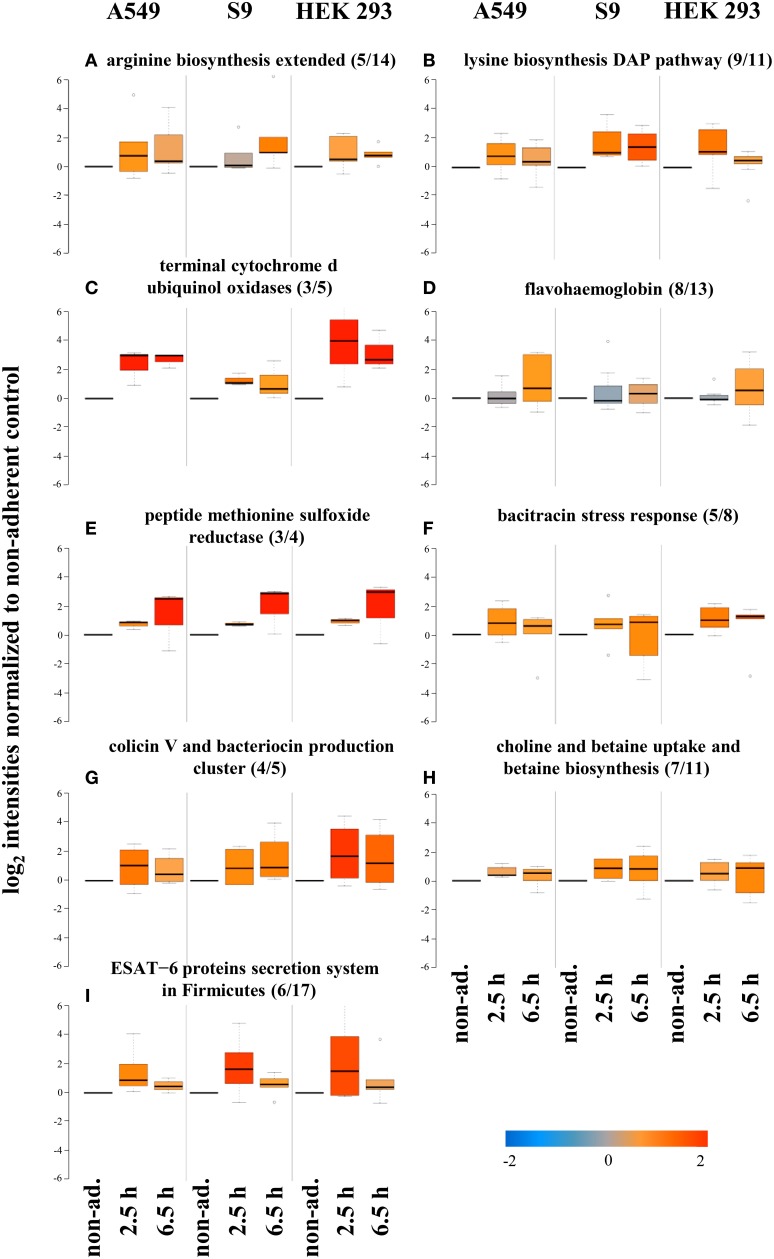
**Protein groups displaying increased levels after internalization by all three cell lines**. Average values of log_2_ intensities from three biological replicates each for non-adherent bacteria as well as 2.5 h and 6.5 h p.i. are depicted. Blue spots indicate lower levels in the internalized bacteria compared to the non-adherent control; red colors represent higher levels of proteins in response to internalization compared to the non-adherent control. **(A,B)** Proteins involved in synthesis of arginine and lysine, **(C)** terminal oxidases, **(D)** flavohaemoglobins, and **(E)** methionine sulfoxide reductase, **(F)** bacitracin stress response, **(G)** colicin V and bacteriocin production, **(H)** choline and betaine uptake, and **(I)** the ESAT-6 secretion system.

Because *S. aureus* also needs to adapt its protein inventory to the special conditions of the intracellular environment, we looked for pathways commonly displaying increased protein levels after entering the different cell lines, compared to non-adherent control bacteria exposed to the same medium and thus nutrient supply conditions. Some amino acid biosynthesis pathways, such as arginine (5 of 14, Figure 6A) and lysine (9 of 11, Figure 6B) biosynthesis, displayed increased protein levels for all cell lines which might be an adaptation to lower amino acids levels inside host cells vs. the cell culture medium. Having the metabolome data at hand, we wanted to make an effort to validate this hypothesis. For A549 cells we could directly compare the intracellular and extracellular concentrations because the cell volume of these cells has previously been reported (Jiang et al., 2010). However, intracellular lysine levels of uninfected host cells were similar to those in the supernatant, and arginine levels could not be measured because of technical reasons. Metabolite levels might differ in infected host cells, but since only a small proportion of host cells indeed carried *S. aureus* we could not assess metabolite levels in this sub-group specifically. Striking differences between the extracellular and host cell concentrations were observed for glycine (intracellular 2.3 mmol/L, extracellular 0.1 mmol/L), threonine (intracellular 4.7 mmol/L, extracellular 0.9 mmol/L), and glutamate (intracellular 16.0 mmol/L, extracellular 0.5 mmol/L) (see Supplementary Material Figure 1).

Levels of subunits of the terminal cytochrome d ubiquinol oxidases (3 of 5) were also higher in internalized *S. aureus* vs. the non-adherent controls (Figure 6C). This observation fits very well to the adaptation of the repertoire of terminal oxidases to the level of oxygen available. While levels of the main oxidase Qox decreased because this enzyme is preferentially used during oxygen excess (see above), the level of the alternative oxidase CydAB increased because this enzyme complex is used as an alternative during microaerobic conditions (Götz and Mayer, 2013), thus supporting the notion of a microaerobic environment inside host cells. Furthermore, increases in the levels of flavohaemoglobin (8 of 13, Figure 6D), peptide methionine sulfoxide reductase (3 of 4, Figure 6E), and bacitracin stress response (5 of 8, Figure 6F) were observed. The same was true for proteins involved in the colicin V and bacteriocin production clusters (4 of 5, Figure 6F) and choline and betaine uptake and betaine biosynthesis (7 of 11, Figure 6G). Also proteins belonging to the ESAT-6 protein secretion system in Firmicutes (6 of 17, Figure 6I) were increased in level upon internalization. These results likely reflect adaptations to the hostile host environment. These observations fit very well to those made for individual cell lines (Schmidt et al., 2010) indicating that these responses are more universal.

#### Activation of the alternative sigma factor SigB upon internalization

**Figure 7:**
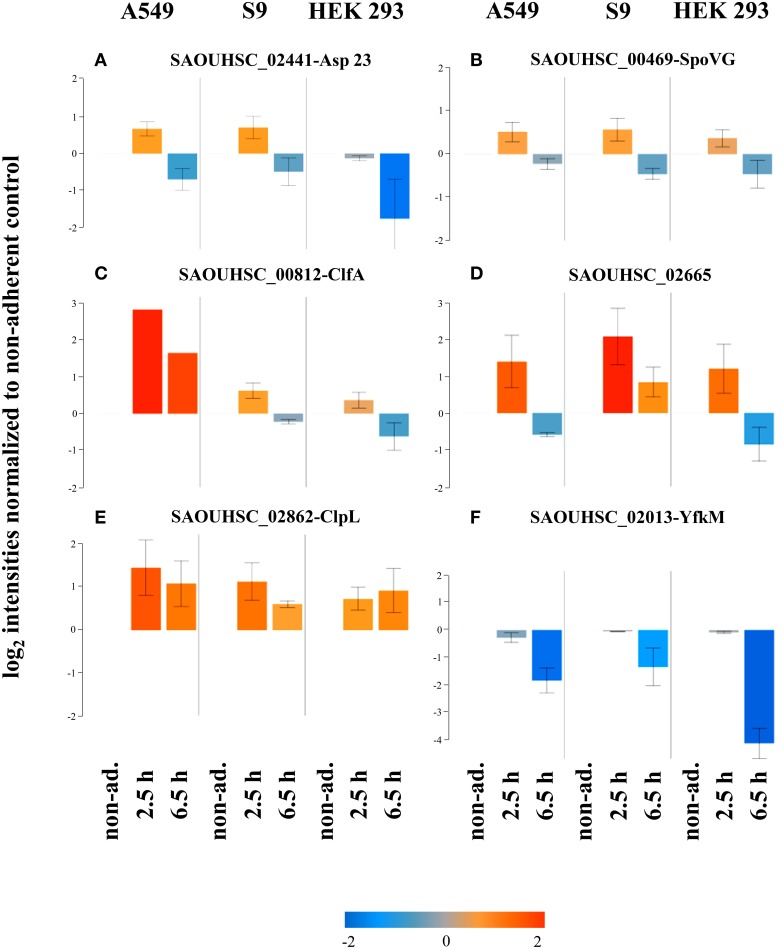
**Activation of SigB following internalization**. Average values of log_2_ intensities from three biological replicates each for non-adherent bacteria as well as 2.5 h and 6.5 h p.i. are depicted for six proteins encoded by members of the SigB regulon. Blue spots indicate lower levels in the internalized bacteria compared to the non-adherent control; red colors represent higher levels of proteins in response to internalization compared to the non-adherent control. **(A)** Asp23, **(B)** SpoVG, **(C)** ClfA, **(D)** SAOUHSC_02665, **(E)** ClpL, **(F)** YfkM.

It has recently been shown that the alternative sigma factor SigB which controls many *S. aureus* genes also with impact on virulence is activated following internalization of *S. aureus* HG001 by S9 cells (Pförtner et al., 2014). Since reports on the role of the alternative sigma factor SigB in different animal models and cell culture settings differ (Jonsson et al., 2004; Depke et al., 2012), we wanted to assess if the activation described for S9 epithelial cells is also conserved upon internalization by A549 and HEK 293 cells. The data presented in Figure 7 support this notion. Of the six proteins for which Pförtner et al. (2014) have clearly shown SigB-dependent increases in level following internalization of *S. aureus* HG001 by S9 cells, five [Asp23 (Figure 7A), SpoVG (Figure 7B), CflA (Figure 7C), ClpL (Figure 7D), and SAOUHSC_02665 (Figure 7F)] displayed increases in level following internalization that were maintained for ClpL even 6.5 h after internalization. The increase in protein content was observed following internalization by all three cell lines, even if the particular patterns sometimes differed. The increase in level could not be confirmed for YfkM (Figure 7F).

#### Differences in the adaptation of *S. aureus* to internalization by different types of host cell lines

In this study, the focus lies on the identification of conserved and specific responses of *S. aureus* HG001 to the three different cell lines, with the HEK 293 cell line constituting an additional host model for the internalization setup in comparison with the two lung epithelial cell lines (S9 and A549). As indicated by the different growth pattern (Figure 1B) and the PCA plot (Figure 3) the strongest differences were observed in the HEK 293 cells.

Here we provide information for selected protein classes and pathways which were differentially regulated depending on the host cell model. Although *S. aureus* HG001 showed similar adaptation patterns over time for all three internalization models, some distinct differences were observed.

**Figure 8:**
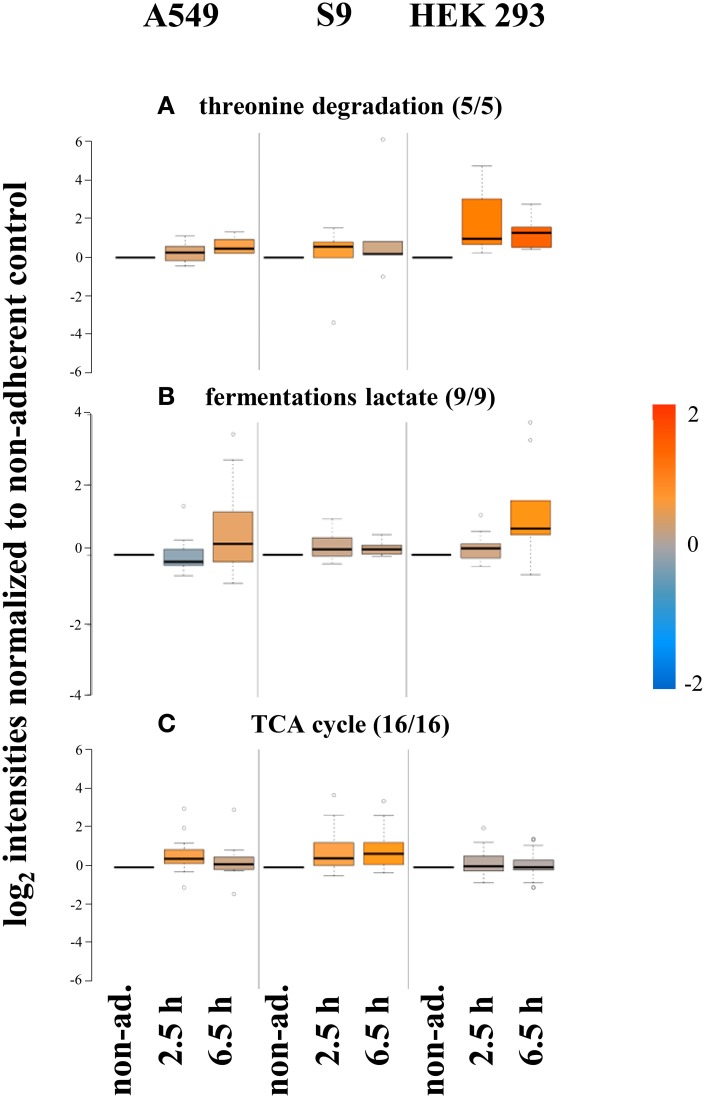
**Proteins differentially regulated 6.5 h p.i. after internalization by different host cells**. Average values of log_2_ intensities from three biological replicates each for non-adherent bacteria as well as 2.5 h and 6.5 h p.i. are represented. Blue spots indicate lower levels in the internalized bacteria compared to the non-adherent control, red colors represent higher levels of proteins in response to internalization compared to the non-adherent control. **(A)** Degradation of threonine is increased inside HEK 293 cells. **(B)** Fermentation of lactate is increased to highest extent inside HEK 293 cells but lowest inside S9 cells. **(C)** Proteins of the TCA cycle are found in lower amounts inside HEK 293 cells.

Different patterns were for example observed for enzymes involved in threonine degradation in HEK 293 cells compared to the two epithelial cell lines (Figure 8A). These differences coincided with differences in the host cell levels of threonine (Supplementary Material Figure 1). The intracellular levels of threonine and the degrading enzymes (5 of 5) were higher in HEK 293 cells. A second example concerns enzymes involved in fermentation (9 of 9), which were present in lower levels in S9 cells compared to *S. aureus* internalized by either A549 or HEK 293 cells (Figure 8B). More specifically, enzymes such as alcohol dehydrogenase (Adh1), L-lactate dehydrogenase (LctE), L-lactate dehydrogenase 2 (Ldh2), and D-lactate dehydrogenase (Ddh) increased in level after internalization by A549 and HEK293 within 6.5 h up to 2-3fold (Ldh2 and Ddh) or 8-10fold (Adh1 and LctE), respectively (Supplementary Material Figure 3A), indicating the supplementary utilization of fermentative enzymes under microaerobic conditions.

The third example is the tricarboxylic acid cycle (TCA). Most of the enzymes of the TCA cycle (16 of 16) that could be monitored in our proteomics approach increased in level in *S. aureus* following internalization by all three cell lines (Figure 8C). This increase probably reflects a switch from glucose to alternative energy-/carbon sources, which depends on increased TCA cycle capacity in internalized bacteria compared to the non-internalized controls. Astonishingly, a number of TCA cycle enzymes including citrate synthase (CitZ), isocitrate dehydrogenase (CitC), succinyl-CoA synthetase subunit beta (SucC) and succinate dehydrogenase flavoprotein subunit (SdhA) increased in level upon internalization by epithelial cells but not in *S. aureus* internalized by HEK293 cells (Supplementary Material Figure 3B).

These selected examples suggest that *S. aureus* can on the one side adapt its physiology to the intracellular life style by conserved adaptation reactions but on the other side also has the capacity to respond to subtle differences encountered in the different host cell niches. Quantitative information about further proteins analyzed in this approach as well as their classification to functional groups is provided as Supplementary Material Tables 1, 2.

## Conclusion

With this study we provide the first time quantitative data for about 50% of the predicted *S. aureus* HG001 proteins from as little as two million internalized *S. aureus* bacteria. The remarkable increase of quantifiable proteins is the result of mass spectrometry applying highly sensitive instruments and utilization of new algorithms for peptide identification. This optimized pipeline allowed us to comprehensively elucidate the early phase of the adaptational response of *S. aureus* after entering human epithelial or kidney cells.

Observations made for specific cell line settings such as the decrease in content of ribosomal proteins or proteins belonging to the *de novo* purine biosynthesis or the increase of proteins belonging to stress responses were now shown to be conserved adaptations reactions to the intracellular environment of human host cells in general.

In addition to common mechanisms of adaptation, we also report differences between *S. aureus* cells internalized by the epithelial cell lines A549 or S9 and the kidney cell line HEK 293, especially 6.5 h p.i., e.g., in threonine degradation as well as level of fermentation enzymes and TCA cycle. Complementing the proteome analysis with metabolomics measurements, we also linked some of the observed adaptation reactions to the altered nutrient supply encountered inside of human host cells. It will now be interesting to extend this studies to extended time courses in which *S. aureus* physiology shifts to the well-known “small colony variants,” a strategy to survive intracellularly (Kahl, 2014) or to study adaptation to survival inside professional phagocytic host cells.

## Author contributions

Kristin Surmann: Design and performance of wet-lab experiments, data analysis, and writing of manuscript. Stephan Michalik: Data analysis and visualization. Petra Hildebrandt: Design and performance of wet-lab experiments. Philipp Gierok: Metabolome analysis, and writing of manuscript. Maren Depke: Data analysis and writing. Lars Brinkmann: Data analysis. Jörg Bernhardt: Data visualization. Manuela G. Salazar: Data acquisition by mass spectrometry. Zhi Sun: Data analysis. David Shteynberg: Data analysis. Ulrike Kusebauch: Data analysis. Robert L. Moritz: Data analysis. Bernd Wollscheid: Data analysis. Michael Lalk: Supervision of metabolome analysis, writing of manuscript. Uwe Völker: Concept and design of experiments, writing of manuscript. Frank Schmidt: Concept and design of experiments, data analysis, and writing of manuscript.

### Conflict of interest statement

The authors declare that the research was conducted in the absence of any commercial or financial relationships that could be construed as a potential conflict of interest.
