# Supplementary material for: Comparative proteome analysis reveals conserved and specific adaptation patterns of Staphylococcus aureus after internalization by different types of human non-professional phagocytic host cells
Source: Front Microbiol. 2014 Aug 1;5:392. doi: 10.3389/fmicb.2014.00392 (PMC4117987; doi:10.3389/fmicb.2014.00392)
Supplement: Supplementary file 1 [file DataSheet1.PDF]

Supplementary Material

Frontiers in Microbiology, Systems Microbiology Original Research 04.07.2014

Comparative proteome analysis reveals conserved and specific adaptation patterns of *Staphylococcus aureus* after internalization by different types of human non-professional phagocytic host cells

Kristin Surmann[1], Stephan Michalik[2], Petra Hildebrandt[2], Philipp Gierok[3], Maren Depke[2], Lars Brinkmann[1], Jörg Bernhardt[4], Manuela Gesell-Salazar[1,2], Zhi Sun[5], David Shteynberg[5], Ulrike Kusebauch[5], Robert L. Moritz[5], Bernd Wollscheid[6], Michael Lalk[3], Uwe Völker[1], and Frank Schmidt[2*]

[1]Interfaculty Institute for Genetics and Functional Genomics, University Medicine Greifswald, Friedrich-Ludwig-Jahn-Str. 15a, 17475 Greifswald, Germany
[2]Interfaculty Institute for Genetics and Functional Genomics, ZIK-FunGene Junior Research Group Applied Proteomics, University Medicine Greifswald, Friedrich-Ludwig-Jahn-Str. 15a, 17475 Greifswald, Germany
[3]Institute of Biochemistry, Ernst-Moritz-Arndt-University Greifswald, Felix-Hausdorff-Straße 4, 17487 Greifswald, Germany
[4]Institute for Microbiology, Ernst-Moritz-Arndt-University Greifswald, Friedrich-Ludwig-Jahn-Str. 15, 17487 Greifswald, Germany
[5]Institute for Systems Biology, 401 Terry Avenue North, Seattle, WA 98109-5234, USA
[6]Institute of Molecular Systems Biology, ETH Zurich, Auguste-Piccard-Hof 1, 8093 Zurich, Switzerland

***Corresponding author's contact information:**

Frank Schmidt, Interfaculty Institute for Genetics and Functional Genomics, ZIK-FunGene Junior Research Group Applied Proteomics, University Medicine Greifswald, Friedrich-Ludwig-Jahn-Str. 15a, 17475 Greifswald, Germany, e-mail: frank.schmidt@uni-greifswald.de, phone: +49-3834-865887, fax: +49-3834-86795871

Settings for nLC-MS/MS analysis

**reversed phase liquid chromatography (RPLC)**	
instrument	Ultimate 3000 RSLC (Thermo Scientific)
trap column	75 µm inner diameter, packed with 3 µm C18 particles (Acclaim PepMap100, Thermo Scientific)
analytical column	25 cm analytical column packed with 2 µm C18 particles (Acclaim PepMap RSLC, Thermo Scientific)
buffer system	binary buffer system consisting of 0.1% acetic acid, 2% ACN (buffer A) and 100% ACN in 0.1% acetic acid (buffer B)
flow rate	300 nl/min
gradient	linear gradient of buffer B from 2% up to 25%
gradient duration	120 min
column oven temperature	40°C

**mass spectrometry (MS)**	
instrument	Q Exactive mass spectrometer (Thermo Scientific)
electrospray	via TriVersa NanoMate (Advion Biosciences, Norwich, UK)
operation mode	data-dependent

**full MS**	
MS scan resolution	70,000
AGC target	3e6
maximum ion injection time for the MS scan	120 ms
scan range	300 to 1650 m/z
spectra data type	centroid

**dd-MS2**	
resolution	17,500
MS/MS AGC target	2e5
maximum ion injection time for the MS/MS scans	120 ms
selection for MS/MS	10 most abundant isotope patterns with charge ≥2 from the survey scan
isolation window	3 *m/z*
fixed first mass	100 m/z
dissociation mode	higher energy collisional dissociation (HCD)
normalized collision energy	27.5%
dynamic exclusion	30 s

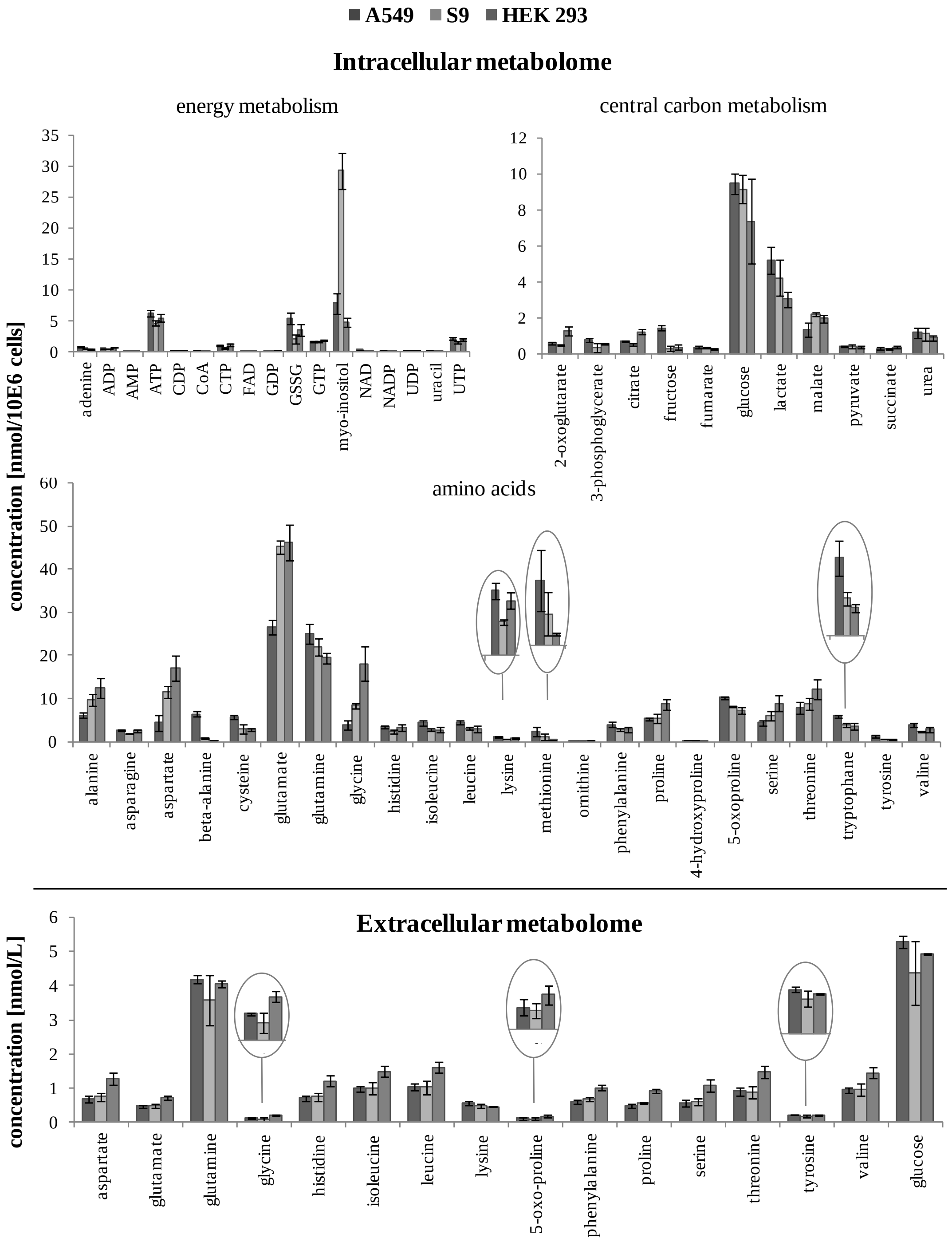

Supplementary Material Fig. 1 Metabolome analysis of host cells. Intracellular metabolites were extracted from non-infected host cells using methanol and measured by GC- and LC-MS. Results are derived from four biological replicates and are given in nmol/10^6 host cells. Most concentrations of metabolites involved in energy metabolism and central carbon metabolism as well as most amino acids were present in similar concentrations in the different host cells. Only some like tyrosine, methionine, citrate or myo-inositol for example differed more than twofold in level for at least one host cell line. Average values and coefficients of variation (CV) can be found as Supplementary Material Table 1. Extracellular metabolites were determined by ^{1}H-NMR from cell culture supernatant of non-infected host-cells from four independent biological samples.

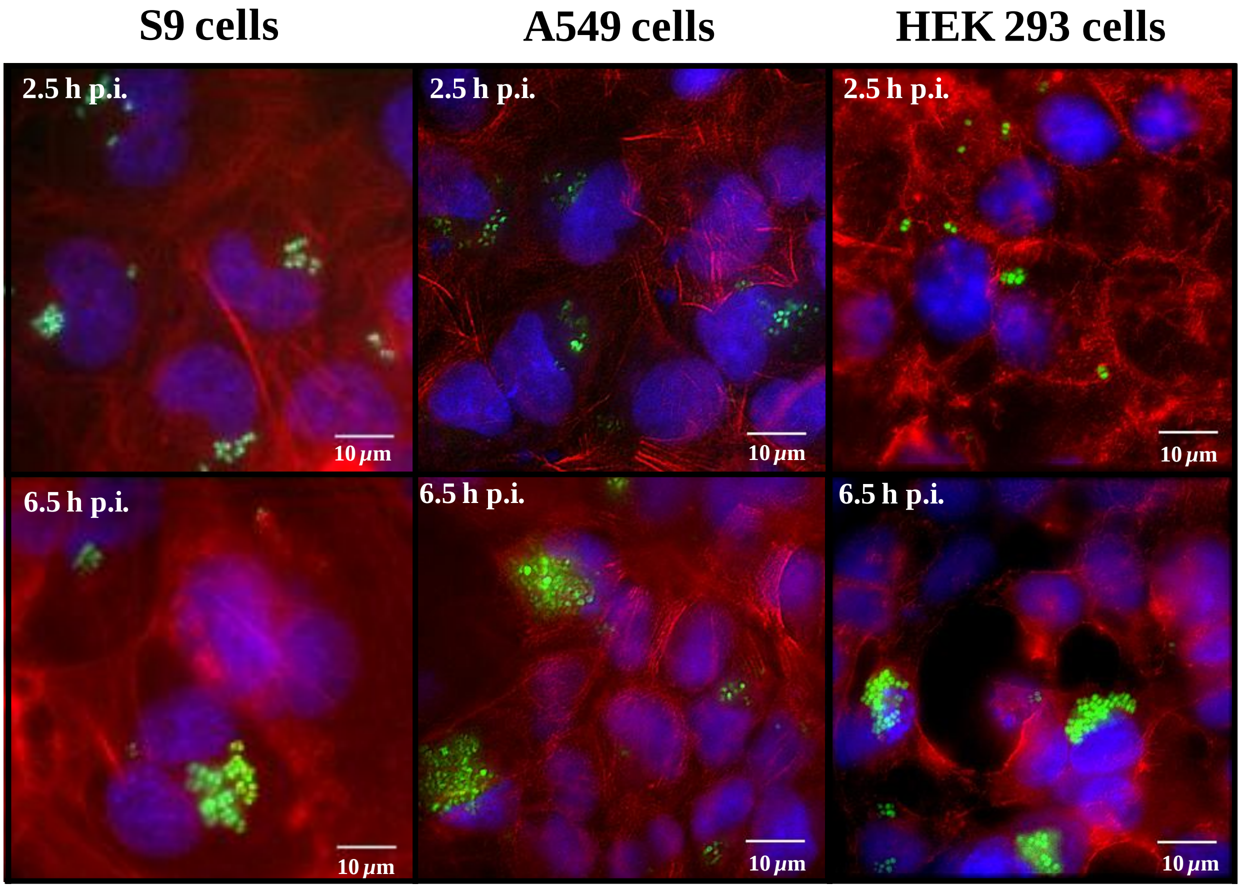

Supplementary Material Fig. 2 Fluorescence pictures of the stained host cell nuclei, F-actin and GFP expressing bacteria with 60x magnification. The size bars indicate 10 μm. The phalloidin-Texas Red stain is shown in red, coloring F-actin. It permits to visualize the contours of eukaryotic cell. The GFP expressing bacteria are visible in green. Hoechst 33258 stains DNA and illustrates the nuclei in blue. Besides an increase in number of bacteria in all three cell lines from 2.5 h p.i. to 6.5 h p.i, also a trend of bacterial cluster formation is detected over time.

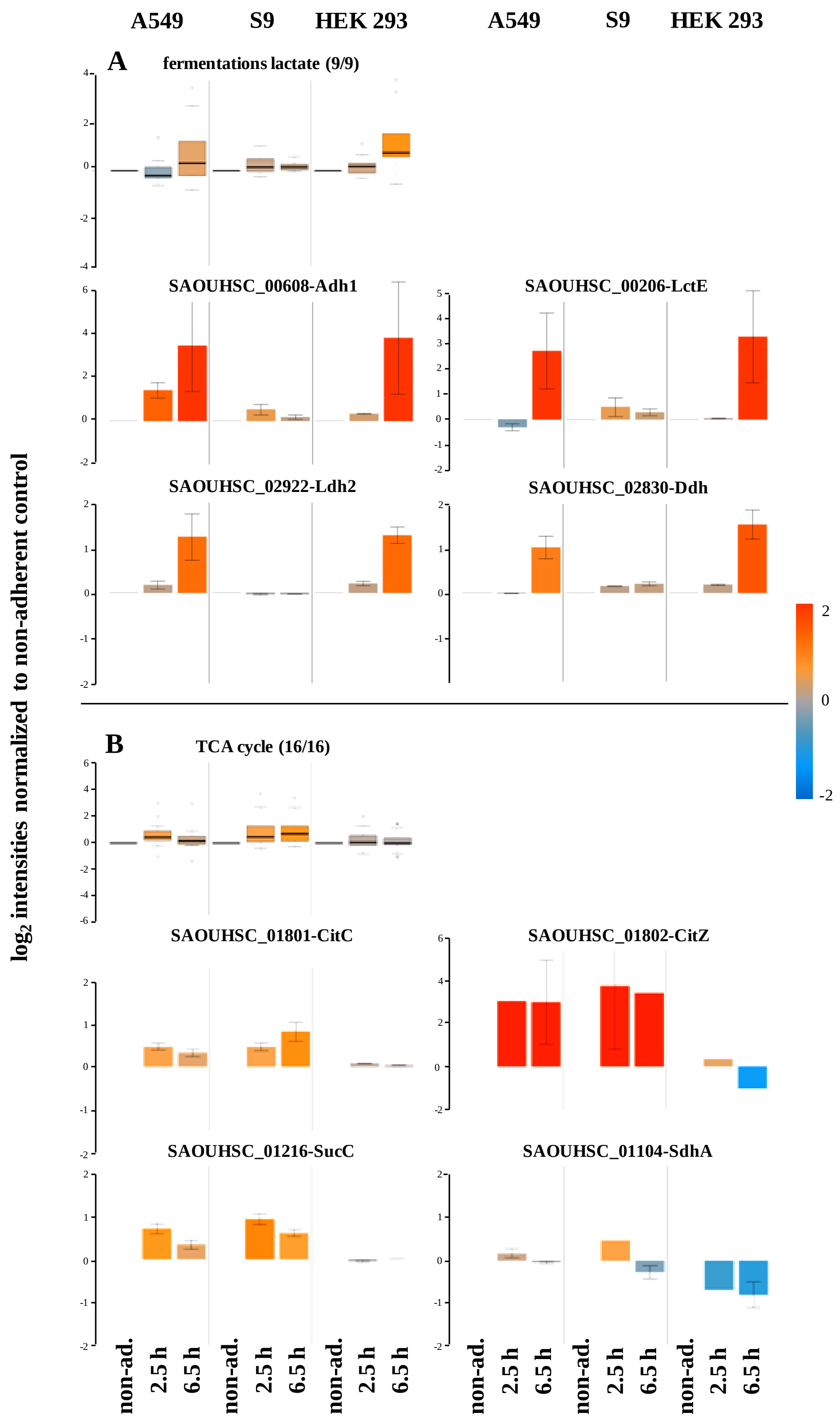

Supplementary Material Fig. 3 Differential regulation of proteins involved in fermentation and TCA cycle after internalization by different cell lines. A) Proteins involved in fermentation are found in lowest amounts inside S9 cells and are induced 6.5 h p.i. inside A549 and HEK 293 cells. B) Proteins of the TCA cycle were found in lower amounts after internalization by HEK 293 cells compared to A549 and S9 cells. Average values of $\log_2$ intensities from three biological replicates each for non-adherent bacteria as well as 2.5 h and 6.5 h p.i. are depicted.

Supplementary Material Table 1 Intracellular metabolome of A549, S9, and HEK 293 cells. Average values and coefficient of variation (CV) from four biological replicates. The energy charge showing the ratios relation between ATP, ADP, and AMP was also calculated for each cell line (A549 0.96, S9 0.95, HEK 293 0.94). According to Atkinson, for living cells this value lies between 0.8 and 0.95 (Atkinson, 1968). Thus, our metabolome analysis occurred with living cells exactly at point of sampling.

	A549 average [nmol/10^6 cells]	A549 CV [%]	S9 average [nmol/10^6 cells]	S9 CV [%]	HEK 293 average [nmol/10^6 cells]	HEK 293 CV [%]
adenine	0.79	13.85	0.54	10.73	0.39	10.04
ADP	0.52	15.22	0.47	7.57	0.70	12.71
AMP	0.03	25.02	0.01	43.70	0.03	18.60
ATP	6.19	9.12	4.66	8.46	5.50	10.64
CDP	0.28	11.35	0.20	7.34	0.18	15.13
CoA	0.20	12.31	0.14	12.17	0.10	15.21
CTP	1.08	13.00	0.63	10.45	1.17	12.56
FAD	0.09	10.55	0.05	7.38	0.04	12.86
GDP	0.11	5.86	0.13	12.06	0.18	8.00
GSSG	5.48	17.36	2.11	34.56	3.44	26.88
GTP	1.64	4.15	1.67	10.03	1.88	9.72
myo-inositol	7.86	21.63	29.29	9.90	4.71	15.72
NAD	0.36	5.44	0.13	2.07	0.16	6.91
NADP	0.20	15.34	0.06	53.86	0.10	32.93
UDP	0.18	11.27	0.17	12.20	0.24	17.43
uracil	0.22	13.93	0.14	31.75	0.09	19.97
UTP	2.21	12.14	1.53	11.26	1.96	10.64
4-hydroxyproline	0.27	12.65	0.20	7.69	0.12	11.05
5-oxoproline	10.18	4.12	8.21	2.16	7.30	9.86
alanine	6.10	10.29	9.71	15.07	12.47	18.74
asparagine	2.67	5.93	1.80	3.88	2.58	10.87
aspartate	4.44	41.10	11.64	12.00	17.05	17.83
beta-alanine	6.40	8.49	0.86	9.28	0.31	20.22
cysteine	5.72	9.35	2.94	32.66	2.93	10.91
glutamate	26.66	6.15	45.22	3.31	46.27	8.89
glutamine	24.95	9.39	22.08	8.79	19.40	6.71
glycine	3.85	27.16	8.36	7.94	18.13	22.59
histidine	3.49	9.46	2.45	17.47	3.17	22.52
isoleucine	4.33	12.84	2.75	12.34	2.71	23.45
leucine	4.54	11.14	3.06	12.18	2.94	21.66
lysine	1.11	12.14	0.57	8.91	0.93	14.88
methionine	2.24	47.85	1.08	69.58	0.39	13.24
ornithine	0.12	11.05	0.11	14.06	0.19	19.69
phenylalanine	4.01	12.68	2.80	8.89	2.80	22.57
proline	5.31	6.17	5.48	19.07	8.67	14.79
serine	4.41	12.60	6.08	18.22	8.83	21.18
threonine	7.81	17.47	8.79	17.10	12.19	19.56
tryptophane	5.91	6.13	4.00	11.63	3.60	19.58
tyrosine	1.35	22.77	0.64	17.49	0.47	14.19
valine	3.81	13.83	2.34	12.01	2.79	24.17
2-oxoglutarate	0.61	11.13	0.46	6.40	1.25	20.24
3-phosphoglycerate	0.76	13.57	0.36	73.95	0.52	7.09
citrate	0.70	8.06	0.51	12.47	1.22	9.91
fructose	1.44	10.96	0.29	47.74	0.37	34.00
fumarate	0.37	14.53	0.33	7.85	0.28	12.64
glucose	9.49	6.04	9.18	8.58	7.37	31.90
lactate	5.23	14.74	4.22	23.72	3.05	13.79
malate	1.34	27.14	2.20	4.10	1.96	11.15
pyruvate	0.42	11.87	0.43	22.99	0.37	20.68
succinate	0.29	16.79	0.26	13.42	0.37	13.48
urea	1.18	25.18	1.10	33.74	0.89	17.01

References

Atkinson, D.E. (1968). The energy charge of the adenylate pool as a regulatory parameter. Interaction with feedback modifiers. *Biochemistry* 7**,** 4030-4034.
